# Alleviating Effect of a Magnetite (Fe_3_O_4_) Nanogel against Waterborne-Lead-Induced Physiological Disturbances, Histopathological Changes, and Lead Bioaccumulation in African Catfish

**DOI:** 10.3390/gels9080641

**Published:** 2023-08-08

**Authors:** Afaf N. Abdel Rahman, Basma Ahmed Elkhadrawy, Abdallah Tageldein Mansour, Heba M. Abdel-Ghany, Engy Mohamed Mohamed Yassin, Asmaa Elsayyad, Khairiah Mubarak Alwutayd, Sameh H. Ismail, Heba H. Mahboub

**Affiliations:** 1 Department of Aquatic Animal Medicine, Faculty of Veterinary Medicine, Zagazig University, Zagazig 44519, Egypt; 2 Department of Forensic Medicine and Toxicology, Faculty of Veterinary Medicine, University of Sadat City, Sadat City 32897, Egypt; basma.elkhadrawy@vet.usc.edu.eg; 3 Animal and Fish Production Department, College of Agricultural and Food Sciences, King Faisal University, P.O. Box 420, Hofuf 31982, Saudi Arabia; 4 Fish and Animal Production Department, Faculty of Agriculture (Saba Basha), Alexandria University, Alexandria 21531, Egypt; 5 Department of Pathology, Faculty of Veterinary Medicine, Zagazig University, Zagazig 44519, Egypt; heba.vet@yahoo.com; 6 Department of Biochemistry, Faculty of Veterinary Medicine, Zagazig University, Zagazig 44519, Egypt; drengyyassin7@gmail.com; 7 Department of Pharmacology, Faculty of Veterinary Medicine, Mansoura University, Mansoura 35516, Egypt; asmaa_ezat@mans.edu.eg; 8 Department of Biology, College of Science, Princess Nourah bint Abdulrahman University, P.O. Box 84428, Riyadh 11671, Saudi Arabia; kmalwateed@pnu.edu.sa; 9 Faculty of Nanotechnology for Postgraduate Studies, Cairo University, Sheikh Zayed Branch Campus, Giza 12588, Egypt; drsameheltayer@yahoo.com

**Keywords:** *Clarias gariepinus*, health status, lead toxicity, magnetite nanogel, nanotechnology, tissue architecture

## Abstract

Heavy metal toxicity is an important issue owing to its harmful influence on fish. Hence, this study is a pioneer attempt to verify the in vitro and in vivo efficacy of a magnetite (Fe_3_O_4_) nanogel (MNG) in mitigating waterborne lead (Pb) toxicity in African catfish. Fish (*n* = 160) were assigned into four groups for 45 days. The first (control) and second (MNG) groups were exposed to 0 and 1.2 mg L^−1^ of MNG in water. The third (Pb) and fourth (MNG + Pb) groups were exposed to 0 and 1.2 mg L^−1^ of MNG in water and 69.30 mg L^−1^ of Pb. In vitro, the MNG caused a dramatic drop in the Pb level within 120 h. The Pb-exposed group showed the lowest survival (57.5%) among the groups, with substantial elevations in hepato-renal function and lipid peroxide (MDA). Moreover, Pb exposure caused a remarkable decline in the protein-immune parameters and hepatic antioxidants, along with higher Pb residual deposition in muscles and obvious histopathological changes in the liver and kidney. Interestingly, adding aqueous MNG to Pb-exposed fish relieved these alterations and increased survivability. Thus, MNG is a novel antitoxic agent against Pb toxicity to maintain the health of *C. gariepinus*.

## 1. Introduction

With the introduction of harmful compounds into the aquatic environment, public health issues connected to environmental pollution are receiving much attention. Heavy metal (HM) pollution is considered one of the most disastrous problems threatening aquatic and human life [1]. Fish are considered a pivotal indicator of aquatic environments for the assessment of the severity of HM toxicity, which constitutes a major hazard for all fish consumers [2,3].

African catfish (*Clarias gariepinus*) has been used to assess HM toxicity. The recent literature reveals the susceptibility of *C. gariepinus* to various HMs and verifies the deleterious impacts of HMs by inducing behavioral changes, immune–antioxidant impairments, and bioaccumulation [4,5,6]. Lead (Pb) is among the most hazardous HMs and is toxic even in low amounts for aquatic animals and humans, resulting in toxic impacts and accelerating different diseases [7]. In aquaculture, exposure to Pb induces oxidative stress, bioaccumulation, neurotoxicity, and immune dysfunction [8]. In *C. gariepinus* and Nile tilapia (*Oreochromis niloticus*), Pb toxicity causes several issues, including hepato-renal toxicity, oxidative damage, histopathological changes, and higher mortality rates [9,10].

Currently, the application of nanomaterials has been proven to have great success in drug delivery, antimicrobial uses, and remediating toxicity caused by either chemical toxicants or HMs in freshwater fishes [11,12,13,14]. Regarding the removal of HMs, engineered nanomaterials represent novel and successful approaches compared to traditional methods. Among the recently formulated nanoparticles, magnetite (Fe_3_O_4_) nanoparticles have interesting electric and magnetic properties and unlimited physical and chemical characteristics at the nanoscale [15,16]. The nano-magnetite form of iron has wide applications in the industry (magnetic recording media, soft magnetic materials, and coloring) and medical sectors (drug delivery, in vivo therapeutic technology, cell separation, and imaging) [17,18]. The magnetite nanocomposites prepared by the sol–gel method have several advantages, including low-cost preparation, toxicity-free iron salts, small particle size, and good dispersion in the solvent [16]. Magnetite nanoparticles (Fe_3_O_4_) succeeded in removing 66% of copper from a solution after 15 min [19]. Magnetite nanoparticles (Fe_3_O_4_ NPs) have been used in *O. niloticus* to chelate mercury (Hg) in vitro, in addition to boosting the immune–antioxidant status and liver and kidney function in vivo [14]. Nanogels (NGs) refer to small, aqueous, swollen nanoparticles composed of nano-scaled polymeric chains [20]. Recently, NGs have emerged as very promising and flexible biomaterials utilized in several applications, such as catalysts, sensing materials, or environmental adsorbents. Their characteristics (such as their wide surface area, flexibility in size, ability to carry molecules, and encapsulation of a high percentage of water when suspended in the fluid) enable their use for drug delivery [21,22]. NGs have been reported in novel environmental fields to eliminate organic toxicants and agrochemicals [23,24]. These contaminants can be trapped inside the NGs, and then removed from the environment [25,26]. In addition, a magnetic nanocomposite sol–gel of iron oxide nanoparticles coated with titanium dioxide efficiently removed aluminum and iron ions from contaminated water [27].

Therefore, this novel study is carried out to investigate the potent magnetic power of a magnetite nanogel (MNG) to mitigate the waterborne toxicity induced by Pb ions via testing their adsorption capacity and, accordingly, testing their magnetic effect to prevent Pb bioaccumulation in muscles. In addition, this study provides an assessment of the promising role of MNG on the protein profile, hepato-renal function, immune responses, tissue antioxidants, and the histological picture of African catfish.

## 2. Results

### 2.1. MNG Characterization

**Figure 1:**
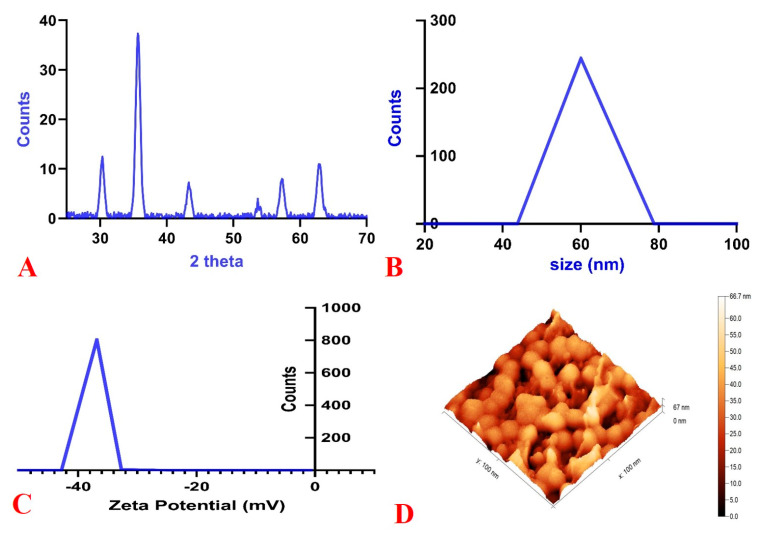
Characterization patterns of magnetite nanogel: (**A**) XRD, (**B**) DLS, (**C**) Zeta potential, and (**D**) AFM.

**Figure 2:**
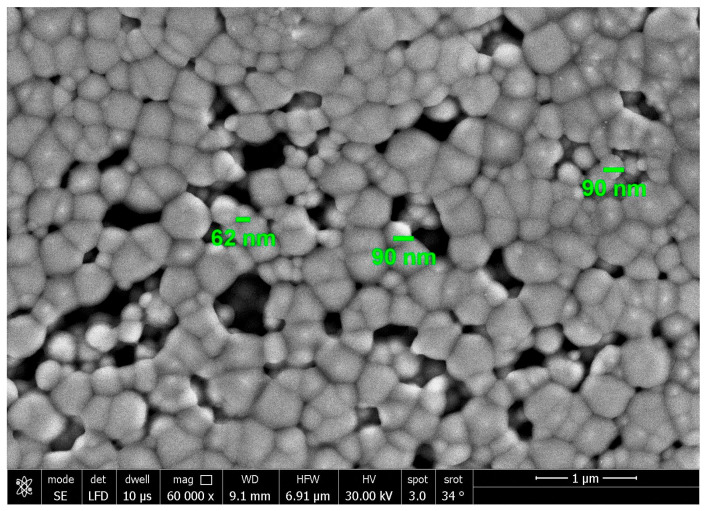
SEM image (1 µm) of magnetite nanogel.

**Figure 3:**
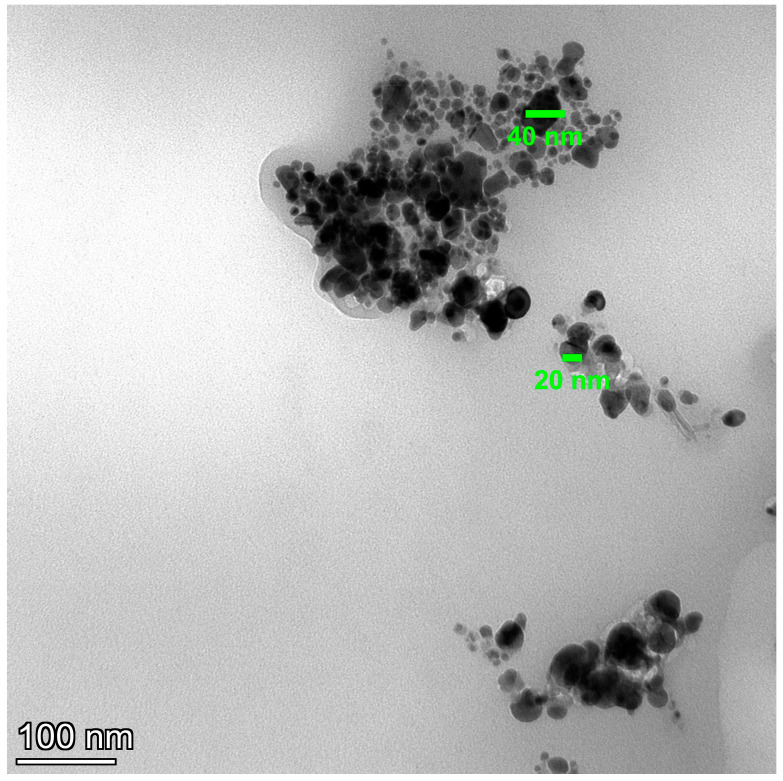
TEM image (100 nm) of magnetite nanogel.

Figure 1, Figure 2 and Figure 3 display various types of MNG characterization findings. X-ray diffraction (XRD) analysis demonstrated the fingerprint curve and data for magnetite according to the Brucker Database library, which conformed to validate our synthesis method without any secondary phases (Figure 1A). Dynamic light scattering (DLS) and zeta potential data showed a homogenous size (one peak) of 60 nm (Figure 1B). Due to a substantial degree of zeta potential (−35 mV), the results demonstrated a superior colloidal structure in aqueous solution (Figure 1C).

Meanwhile, gel formation had no characteristic peaks due to its amorphous nature. The morphology illustrated by atomic force microscopy (AFM), scanning electron microscopy (SEM), and transmission electron microscopy (TEM) showed the spherical shape of MNG (Figure 1D, Figure 2, and Figure 3).

### 2.2. Absorption of Pb Ions by MNG

**Figure 4:**
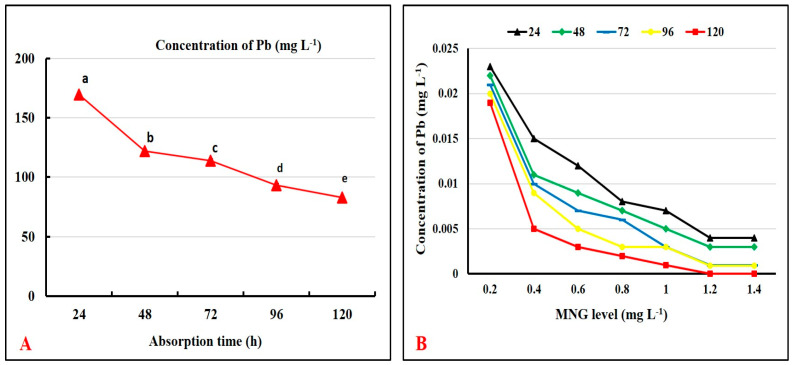
(**A**) Absorption of lead (Pb) by magnetite nanogel (MNG) across 24, 48, 72, 96, and 120 h. (**B**) Impact of MNG level on the concentration of Pb ions across 24, 48, 72, 96, and 120 h. Values that did not have the same superscripts differ significantly (one-way ANOVA; *p* < 0.05).

Figure 4A shows that MNG caused a dramatic drop in the concentration of Pb ions throughout all sampling points. The concentration decreased from 169.53 mg L^−1^ at the beginning of the experiment to 82.87 mg L^−1^ after 120 h.

The various MNG concentrations affected the elimination of the Pb ions, as seen in Figure 4B. The findings showed that raising the MNG level lowered the amount of Pb ions in the aquarium water and allowed for the removal of reduced Pb metal. The outcomes also showed that 1.2 and 1.4 mg/L of MNG were the ideal doses that produced the greatest Pb ion adsorption loading.

### 2.3. Mortality and Clinical Observations

**Figure 5:**
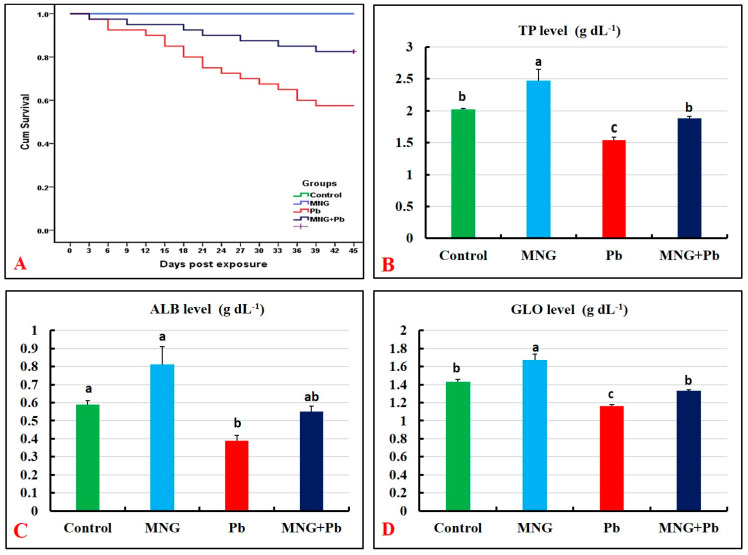
Cumulative survival (*n* = 40/group) and protein profile parameters (*n* = 12/group) of *C. gariepinus* exposed to magnetite nanogel (MNG) and/or lead (Pb) as a water exposure for 45 days. (**A**) Survival curves (Kaplan–Meier). (**B**) Total proteins (TP). (**C**) Albumin (ALB). (**D**) Globulins (GLO). Bars (means ± SE) that did not have the same superscripts differ significantly (one-way ANOVA; *p* < 0.05).

Based on Kaplan–Meier curves (Figure 5A), the survival rate was 100% in the control and MNG groups during the experimental period (45 days). The lowest survival rate was recorded in the Pb group (57.5%). There was a marked elevation in the survival rate in the MNG + Pb group (82.5%) compared with the Pb group.

The clinical examination of the treated fish showed that neither the control nor the MNG groups exhibited any atypical behaviors or disease symptoms during the 45 days of exposure. On the contrary, the fish of the Pb group featured symptoms of respiratory distress manifested by rapidly moving the operculum and air gasping from the surface. Fish also developed a slimy appearance, severe skin rot, darkening, and erosions with hemorrhages. Internally, the gills were pale with congestion of internal organs. The Pb and MNG (MNG + Pb)-exposed group showed a remarkable return to the typical appearance with minimal fin rot and a mildly congested liver.

### 2.4. Hepato-Renal Function Biomarkers

Table 1 demonstrates no discernible variations in the values of hepato-renal biomarkers (ALT, AST, ALP, creatinine, and urea) between the MNG and control groups. These biomarkers displayed the highest values (*p* < 0.05) in the Pb group compared to the control. In contrast, treatment of Pb-exposed fish with MNG resulted in a significant decrease (*p* < 0.05) in these variables compared to Pb exposure alone.

### 2.5. Protein Profile and Immune Status

**Figure 6:**
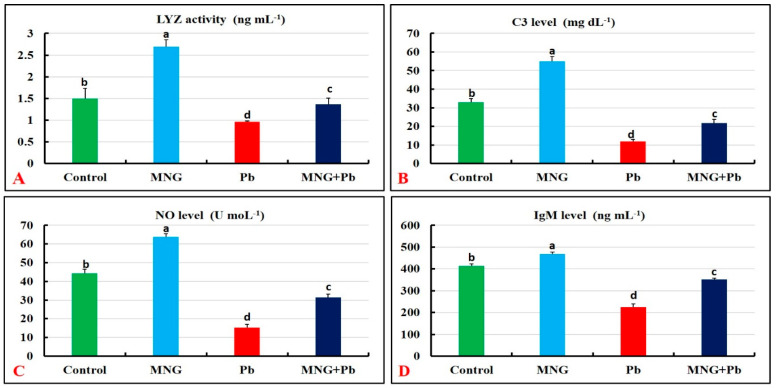
Immune parameters of *C. gariepinus* exposed to magnetite nanogel (MNG) and/or lead (Pb) as a water exposure for 45 days (*n* = 12/group). (**A**) Lysozyme activity (LYZ). (**B**) Complement 3 (C3). (**C**) Nitric oxide (NO). (**D**) Immunoglobulin M (IgM). Bars (means ± SE) that did not have the same superscripts differ significantly (one-way ANOVA; *p* < 0.05).

Figure 5B–D and Figure 6A–D demonstrate substantial augmentations (*p* < 0.05) in the protein profile (TP, ALB, and GLO) and immune (LYZ, C3, NO, and IgM) parameters in the MNG group related to the control. Meanwhile, the lowest concentrations of these biomarkers were observed in the Pb-exposed fish, followed by the MNG + Pb fish.

### 2.6. Hepatic Oxidant/Antioxidant Status

Table 2 shows the levels of MDA and antioxidants in the liver (GSH, SOD, and CAT) of *C. gariepinus* after the exposure period (45 days). There was no noticeable variation in the MNG group’s MDA level compared with the control one; however, a significant elevation (*p* < 0.05) in the GSH, SOD, and CAT values was noticed. Pb exposure induced a profound elevation in the MDA level and lessened the antioxidant values relative to the control. The values of these variables showed more improvement in the MNG + Pb group than in the Pb group.

### 2.7. Histopathological Findings

**Figure 7:**
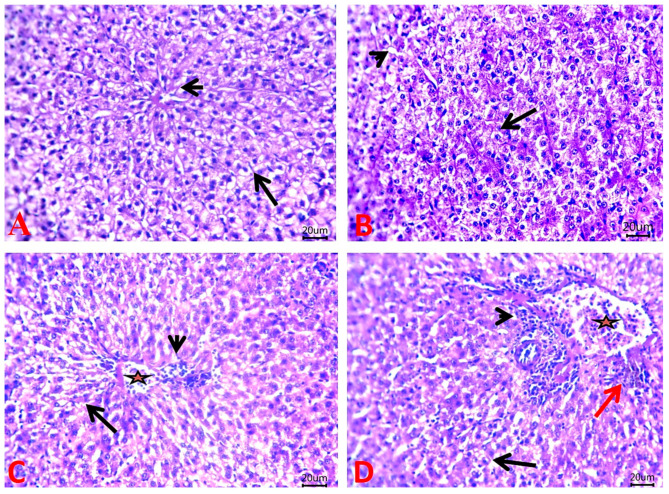
Photomicrograph of H&E-stained liver sections of *C. gariepinus* exposed to magnetite nanogel (MNG) and/or lead (Pb) as a water exposure for 45 days. (**A**,**B**) Liver of the control and MNG groups, respectively, showing normal histological structures of hepatic acini (arrow) and vasculatures (arrowheads). (**C**) Liver of the Pb group showing a focal area of fatty change (arrow), congested hepatic blood vessel (star), and perivascular inflammatory cell infiltrates (arrowhead). (**D**) Liver of the MNG +Pb group showing microvacuoles within a few numbers of hepatocytes (arrow), congested hepatic blood vessels (star), inflammatory cell aggregate within the portal area (arrowhead), and perivascular aggregation of melanomacrophage (red arrow). Scale Bar: 20 μm.

According to the histopathological investigations, the livers of the control and MNG fish both displayed normal histological structures of hepatic acini and vasculatures (Figure 7A and Figure 7B, respectively). On the contrary, the Pb exposure caused areas of fatty changes, congested hepatic blood vessels, and perivascular inflammatory cell infiltrates (Figure 7C). The livers of the MNG + Pb group exhibited an improvement of lesions as depicted by the appearance of microvacuoles within a small number of hepatocytes, congested hepatic blood vessels, inflammatory cells aggregated within the portal area, and perivascular aggregation of melanomacrophage (Figure 7D).

**Figure 8:**
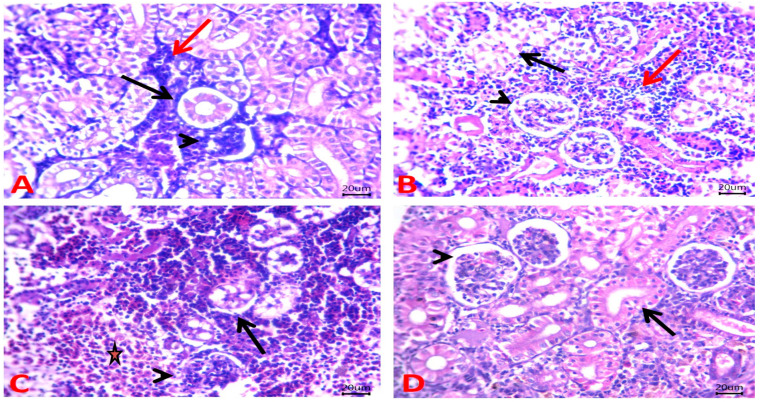
Photomicrograph of H&E-stained kidney sections of *C. gariepinus* exposed to magnetite nanogel (MNG) and/or lead (Pb) as a water exposure for 45 days. (**A**,**B**) Kidney of the control and MNG groups, respectively, showing normal renal structures with preserved glomerular capillary tufts (arrowheads), renal tubular epithelium (arrows), and the presence of hemopoietic cells (red arrows). (**C**) Kidney of the Pb group showing marked necrotic changes in the tubular epithelium (arrow), maintained glomerular architectures (arrowhead), and depletion of hemopoietic center replaced by pale eosinophilic substance (star). (**D**) Kidney of the MNG + Pb group showing normal histomorphological structures of the renal tubule (arrow) and glomerular corpuscle (arrowhead). Scale Bar: 20 μm.

Moreover, normal renal structures with preserved glomerular capillary tufts, renal tubular epithelium, and hemopoietic cells were clear in the fish kidney of the control and MNG groups (Figure 8A and Figure 8B, respectively). However, Pb exposure induced histopathological alterations in the kidney, which appeared as marked necrotic changes in tubular epithelium and maintained glomerular architectures. Further, a depletion of the hemopoietic center replaced by a pale eosinophilic substance was obvious (Figure 8C). Treatment of Pb-exposed fish with MNG markedly improved these alterations and revealed normal histopathological structures of renal tubules and glomerular corpuscles (Figure 8D).

### 2.8. Bioaccumulation of Pb^2+^ in Fish Muscles

**Figure 9:**
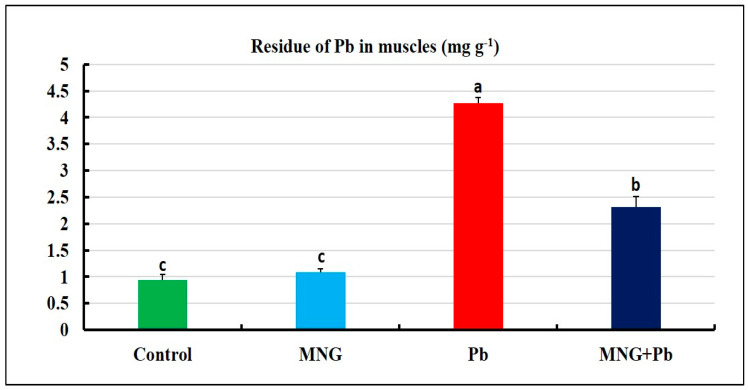
Residues of lead (Pb) in muscles of *C. gariepinus* exposed to magnetite nanogel (MNG) and/or Pb as a water exposure for 45 days (*n* = 12/group). Bars (means ± SE) that did not have the same superscripts differ significantly (one-way ANOVA; *p* < 0.05).

The concentration of Pb ions in the muscles of the MNG and control groups did not alter significantly (*p* > 0.05), as presented in Figure 9. The muscles of the Pb group had the highest levels of Pb ions. Still, the MNG + Pb group had considerably lower levels of Pb residues.

## 3. Discussion

The toxicity of Pb has a slow-acting cumulative impact that results in major health problems for aquatic animals and humans because of its use in various industrial processes that contaminate water [28]. The current report is an innovative trial to underpin the effectiveness of MNG to alleviate the toxicity of Pb in vitro and in vivo via assessing its magnetic power to protect fish muscles from Pb bioaccumulation and studying its potential role on protein picture, hepato-renal function, immune-antioxidant response, and tissue architecture in African catfish.

Among heavily studied nanoparticles, the magnetite nanoscale has attracted more interest owing to the potent power of magnetite to adsorb heavy metal ions in solutions. The nanosized Fe_3_O_4_ particles remove heavy metals via their magnetic properties, high surface area, chemical stability, easy synthesis, and low toxicity [29]. Pb was chosen for testing the adsorption capacity of MNG because it is one of the most predominant dangerous HMs in aquaculture practice [30]. Our findings showed that MNG had a potent adsorbing power of Pb ions that caused a clear reduction in Pb ions with time (120 h). Also, the best adsorption loading was provided by 1.2 and 1.4 mg L^−1^ MNG concentrations relative to the other concentrations. The adsorption power was raised at the start of the experiment and then declined by progressing the adsorption time. These findings could be related to the adsorption and the decrease of the Pb^2+^ ions to Pb metal on the surface of the MNG until the saturation of the MNG surfaces by the Pb ions. The magnetite has a specific crystal structure consisting of free electrons (Fe^2+^ and Fe^3+^) conjugated with oxygen. In the crystal structure, the localization of free electrons [31] is responsible for the magnetic properties, which elevate the surface activities and the adsorption power of the magnetite to lessen the Pb ions on the surface of the MNG. The sorption reaction between magnetite and Pb is chemical adsorption [32]. In the same manner, Hong et al. [33] verified the efficacy of MNG in getting rid of more than 80% of Pb, chromium (Cr), and cadmium (Cd) from contaminated water at 1 mg L^−1^ because of the emerging electrostatic attraction between the positive metal ions and the negatively charged ions of iron oxide.

Considering the clinical picture and post mortem examination, exposure to Pb alters the general health of *C. gariepinus*. Additionally, fish suffered anorexia, major signs of respiratory manifestation, profound mucous secretion production, fin rot, severe erythema, erosion in the skin, and the lowest survival rate (57.5%). It is assumed that the Pb ions irritate the skin and gills because of their direct contact with fish in the aquatic environment, inducing respiratory distress and erythema with more mucous production as a defense reaction against the toxic Pb ions. Our findings were concurrent with those of Alfakheri et al. [34] and Abdel Rahman et al. [10], who noted that the exposure of *C. gariepinus* and *O. niloticus* to Pb toxicity induces respiratory problems and mortalities. On the other hand, exposure to MNG improved the clinical picture and reduced the mortalities in Pb-exposed fish. It is assumed that there are two reasons: the first is the potent magnetism of the magnetite, which enables MNG to adsorb the Pb ions, resulting in a decrease in its level. The other reason is the verified potent antioxidant activity of the NGs, which counteracts the oxidative damage produced by Pb ions. Likewise, Mahboub et al. [14] recorded no mortalities in the mercury-exposed *O. niloticus* with Fe_3_O_4_ NPs.

LYZ, complement activity, NO, and IgM are non-specific and important components that mainly indicate innate immunity in fish [35,36]. TP indicates activated humeral immunity in aquatic organisms [37]. Herein, we reveal the occurrence of immune suppression upon exposure to Pb reflected by a clear reduction in immune parameters, including lysozymes, C3, NO, IgM, and TP. Concurrent with an earlier study, Shah [38] recorded that lethal and even sub-lethal exposure to Pb alters the immunological biomarkers in tench (*Tinca tinca*). Likely, Alandiyjany et al. [12] found a clear depression in the level of TP following the exposure of *O. niloticus* to Pb.

On the other hand, immunomodulation has been reported upon the exposure of *C. gariepinus* to aqueous MNG, which is indicated by a noticeable increase in the immunological biomarkers.

The mechanism of action of Fe_3_O_4_ NPs on the immune system was recently documented by Huang et al. [39], who revealed that the degradation products of the magnetic nanoparticles improve immune stimulation via the interferon gene activating protein (STING) pathway, which, in turn, enhances cellular immune response. A similar report found that Fe_3_O_4_ NPs had an immunological influence by augmenting the activity of LYZ in *O. niloticus* exposed to mercury toxicity [14].

Detoxification of HMs is mainly carried out in the hepatic tissue, followed by filtration and excretion in the renal tissue. Hence, elevating the concentration of HMs induces an increased rate of filtration and detoxification in the fish body, which in turn causes hepato-renal dysfunction [40]. In the present investigation, the biomarkers of renal functions (creatinine and urea) and hepatic enzymes (ALT, ALP, and AST) exhibited an elevation in their levels upon exposure to Pb. It is assumed that Pb induces necrosis in the liver, and accordingly, this damage leads to the leakage of hepatic enzymes into the bloodstream, producing an elevation. Furthermore, Pb toxicity impairs renal function by minimizing its ability to excrete urine, urea and by impairing the glomerular filtration rate, as Akturk et al. [41] described. These attributions were confirmed by the histopathological alteration that was observed in the liver and kidney in our study. In line with the present findings, Abdel-Tawwab et al. [42] revealed that a noticeable increase in the values of urea and creatinine was recorded in *O. niloticus* after intoxication with a mixture of HMs, including Pb. Histological alterations of the liver and gill tissue of *C. gariepinus* were reported post-exposure to Pb, including fibrosis of hepatic cords and necrosis of parenchyma cells besides collapsing blood vessels [43].

On the other side, a restoration of hepato-renal biomarkers in the aqueous MNG + Pb-treated group and a clear regeneration of histological changes indicated the protective effect of MNG against Pb-induced hepato-renal damages. It is suggested that aqueous MNG can mitigate the hazardous effects of Pb toxicity by lessening the Pb-generated reactive oxygen species (ROS) on hepatic cells. Similarly, Mahboub et al. [14] reported that Fe_3_O_4_ NPs had a promising effect on improving hepato-renal functions of *O. niloticus* and could enhance the levels of liver enzymes and renal parameters upon exposure to mercury toxicity. A recent study conducted by Alandiyjany et al. [12] reported severe histopathological changes in the liver and gills of *O. niloticus* following exposure to Pb, and a noticeable improvement was detected in the magnetized silica-received group.

HMs induce oxidative damage by generating ROS. The antioxidant defense mechanism involves various enzymes, such as CAT, SOD, and GSH, which protect cells from oxidative stress by detoxifying ROS [44]. The current work showed that oxidative damage in the Pb-exposed group reflected a clear elevation in MDA level and a reduction in GSH, CAT, and SOD. It is opined that Pb causes excess production of ROS, resulting in oxidative damage. In line with recent work, Alandiyjany et al. [12] detected decreased serum CAT, SOD, and GSH activity levels in Pb-exposed *O. niloticus*.

Contrarily, the exposure of fish to MNG in the Pb-exposed group has an antioxidant-protecting effect indicated by a clear modulation in the antioxidant biomarkers (elevated SOD and CAT activities) resulting in protection from oxidative damage. In line with a recent finding, Răcuciu et al. [45] confirmed that Fe_3_O_4_ NPs have potent antioxidant enzymatic activity via modulating the levels of CAT and SOD and aid in plant development. Moreover, Fe_3_O_4_ NPs can enhance the antioxidant status and reduce the oxidative stress of *O. niloticus* and Indian major carp (*Labeo rohita*) [14,46,47].

HM toxicity produces variable immunological and physiological responses, allowing for the bioaccumulation of metals in different fish tissues [7]. Here, we find that the Pb-exposed group’s muscles have a greater level of Pb. In line with a recent report, Alandiyjany et al. [12] detected bioaccumulation of Pb in the muscles of *O. niloticus* following exposure to Pb.

In contrast, the MNG + Pb group reflected the least accumulation of Pb, indicating its efficacy in removing Pb. It is assumed that the magnetic power of magnetite found in MNG, plus the formulation of NGs, enables it to absorb Pb strongly. Previous studies supported our outcomes and documented that the structure of NGs causes them to be easily biocompatible and biodegradable and can absorb and release molecules for decontaminating water, catalysis, and sensors [48,49]. Furthermore, Neamtu et al. [50] added that NGs can absorb active materials through chemical interactions such as hydrogen or hydrophobic bonding and salt formation. Similar outcomes were observed by Alandiyjany et al. [12] in the muscles of *O. niloticus.*

## 4. Conclusions

The present study demonstrates that Pb is a hazardous heavy metal that causes a decline in the survival rate, suppresses immune-antioxidant status, and deteriorates hepato-renal functions and histopathological structure of the liver and kidney tissues. Also, Pb exposure results in high bioaccumulation in the muscles of the treated African catfish. The basic attention is directed to the magnetic antitoxic power of MNG to adsorb Pb ions and protect fish from bioaccumulation in muscles. Additionally, MNG enhances the immune-antioxidant profile, improves the hepato-renal function, and regenerates the histopathological picture. Further studies are mandatory to assess other applications of MNG in various fish species and to assess the safe use on a large scale for sustaining aquaculture and maintaining human health.

## 5. Materials and Methods

### 5.1. Synthesis and Characterization of MNG

Firstly, Fe_3_O_4_ NPs were synthesized following the protocol of Hamdy et al. [51]. About 0.4 g of the hematite ore (Fe_3_O_4_) was added drop by drop to 40 mL of H_2_O_2_. At the same time, the mixture was subjected to ultrasound at 60 kHz for 2.5 h in an ultrasonic device (Sonica 4200 EPS3, Milano, Italy) until the black particles of Fe_3_O_4_ were obtained. After 1.5 h, the Fe_3_O_4_ NPs (black color) precipitated from the supernatant (reddish color). The Fe_3_O_4_ NPs were separated from the solution by centrifugation at 4000 rpm, and, finally, the Fe_3_O_4_ NPs were washed four times using methanol.

For the synthesis of Fe_3_O_4_ NPs/carbopol hybrid nanogel, 0.2 g of Fe_3_O_4_ NP desperation in 25 mL of ethanol was added to a solution of 0.25 g carbopol dissolved in 25 mL of ethanol and the mixture was stirred using a mechanical stirrer for 50 min. Then, 0.75 mL of trimethylamine was added drop by drop and stirred for another 40 min until obtaining a black gel. The Fe_3_O_4_ NPs/carbopol hybrid nanogel was prepared in high and low viscosities.

Characterization protocols were categorized into three groups: morphology, identification, and index class, according to the Hassan et al. [52] approach.

### 5.2. Preparation of Pb Ion Solution

In this experiment, lead chloride (PbCl_2_; purity 98%) of Merck, Darmstadt, Germany was utilized as a source of Pb ions. To reach the proper concentrations, PbCl_2_ was primarily dissolved in de-ionized water to create a stock solution (1000 mg L^−1^) which was then diluted to the necessary concentration before being used in aquarium water. According to Alfakheri et al. [34], the 96 h median lethal concentration (LC_50_) for Pb was 231 mg L^−1^ and 30% of 96 h LC_50_ (69.30 mg L^−1^) was used.

### 5.3. Adsorption Capacity of MNG

In two different studies, the capacity of MNG to adsorb the Pb ions was evaluated. In the first experiment, at 24 °C and pH 6.0, an exact amount of PbCl_2_ (20 mg) was mixed with 100 mL of ultrapure water. In a glass vial, 20 mL of prepared PbCl_2_ and 20 mg of MNG were mixed and vortexed for 10 min to assess the adsorption kinetics per the Kôsak et al. [53] technique. Daily, for five days (24, 48, 72, 96, and 120 h), and using an atomic absorption spectrophotometer (Buck Scientific, Norwalk, CT, USA), the concentration of Pb^+2^ ions was calculated. Three copies of each sample were tested.

The second experiment examined how varied MNG concentrations (0.2, 0.4, 0.6, 0.8, 1, 1.2, and 1.4 mg L^−1^) affected the adsorption ability of Pb ions. It involved setting up seven aquariums with water in them, adding 0.025 mg L^−1^ of Pb ions to each aquarium at pH 6.0, and then adding the seven concentrations of MNG directly to each Pb-exposed aquarium [4]. Then, using the atomic absorption spectrophotometry technique, the level of Pb ions was assessed after 24, 48, 72, 96, and 120 h. The safe recommended level of iron in fish, which varies between 0.35 and 1.7 mg/L [54,55], was considered when choosing the concentrations of MNG.

### 5.4. Ethical Agreement and Fish Acclimation

The Institutional Animal Care and Use Committee of Zagazig University in Egypt (ZU-IACUC/2/F/309/2022) approved the experimental strategy. Two hundred and forty African catfish (100 ± 7.39 g) were selected from the Al-Abbassa private fish farm in Sharkia Governorate, Egypt. The fish were kept for ten days in 100 L of well-aerated aquaria for acclimation. Part of the water was partially exchanged (25%). The fish were supplemented with a basal diet at 3% of their body weight twice daily during acclimation and experimental trial. Assessment of physio-chemical parameters of the rearing water was carried out daily, including temperature, dissolved oxygen, ammonia, and pH, and recorded as 24 ± 2 °C, 6 ± 0.26 mg L^−1^, 0.01 ± 0.04 mg L^−1^, and 7 ± 0.13, respectively.

### 5.5. Assessing the Initial Concentration of MNG

Fish (*n* = 80) were exposed to 8 various concentrations of MNG for 15 days (Table 3) to determine the starting concentration for the treatment experiment. These concentrations were 0, 0.2, 0.4, 0.6, 0.8, 1, 1.2, and 1.4 mg L^−1^ of MNG. The clinical observations were kept track of every day during the preliminary trial. MNG concentrations were safe in the 0.2 to 1.4 mg L^−1^ range, and 1.2 mg L^−1^ was determined to be the dose used for treatment.

### 5.6. Experimental Design

For 45 days, fish (*n* = 160) were randomly assigned into four groups (10 fish/replicate; 40/group). The first and second (MNG) groups were exposed to 0 and 1.2 mg/L MNG in water, where the control was the first group. The third (Pb) and fourth (MNG + Pb) groups were exposed to 0 and 1.2 mg L^−1^ MNG in water, respectively, and 69.30 mg L^−1^ of lead chloride. Fish were moved to freshly produced solutions with the same concentrations daily for 45 days during the experiment. Every day, about 25% of the aquarium’s contents were replenished. Clinical observation and mortalities were kept track of throughout the trial.

### 5.7. Sampling

Fish were randomly selected (12 fish per group) at the end of the experiment (45 days) to collect samples. According to Neiffer and Stamper’s [56] approach, fish were anesthetized with a benzocaine solution (100 mg L^−1^), and blood was then drained from the caudal blood vessels using tubes devoid of the anticoagulant. Samples were centrifuged at 1750× *g* for 10 min after being incubated at room temperature (21 ± 3 °C) for 5 h. Clear serum was then kept at 20 °C until biochemical and immunological assays. Liver tissues (12 fish/group) were gathered and kept in liquid nitrogen for the oxidant/antioxidant assay. Additionally, liver and kidney samples (12 fish/group) were used for histopathology analysis, and samples of muscles (12 fish/group) were picked for determining Pb residues.

### 5.8. Evaluation of Hepato-Renal Function Biomarkers

The activity of hepatic function biomarkers, including aspartate aminotransferase (AST, Catalog No.; EK12276) (Biotrend Co., Laurel, MD, USA), alanine aminotransferase (ALT, Catalog No.; MBS038444) (MyBioSource Co., CA, USA), and alkaline phosphatase (ALP, Catalog No.; TR11320) (Thermo Fisher Scientific, Swindon, UK) were computed. Also, the total protein (TP, Catalog No.; MBS9917835), albumin (ALB, Catalog No.; MBS019237), and urea (Catalog No.; MBS9374784) (MyBioSource Co., CA, USA) were measured. All the biomarkers mentioned above were computed using a spectrophotometer (Lambda EZ201; Perkin Elm, Beaconsfield, UK). The globulin (GLO) level was determined by subtracting ALB from TP. The creatinine (Catalog No.; MAC080) level was estimated at a wavelength of 340 nm using a spectrophotometric protocol (Centromic Gmbit kit manual, Wartenberg, Germany).

### 5.9. Immune Assays

The immune parameters, including lysozyme activity (LYZ), were estimated using the inhibition zone method in agarose gel plates, depending on the protocol of Lee and Yang [57]. The level of complement 3 (C3) was measured by immunoturbidimetry using the method of Abdollahi et al. [58] with separated Eastbiopharm ELISA kits (Hangzhou Eastbiopharm CO., LTD., Torrance, CA, USA).

To quantify the serum nitric oxide (NO), about 100 mL of each serum sample was added to the Griess reagent, which was then incubated for 10 min at 27 °C [59]. Immunoglobulin M (IgM) was quantified in serum spectrophotometrically using ELISA kits (Cusabio Biotech Co., Ltd., Wuhan, China) as directed by the manufacturer, following Schultz’s [60] approach.

### 5.10. Hepatic Oxidant/Antioxidant Assays

According to the Siroka et al. [61] assay, the liver samples were prepared to estimate the levels of oxidant/antioxidant biomarkers (malondialdehyde (MDA), reduced glutathione content (GSH), catalase (CAT), and superoxide dismutase (SOD)). Liver samples were subjected to homogenization in a buffer with a pH of 7.5 to obtain the supernatant, which was then obtained by centrifuging them at 4 °C for 15 min at 10,000 × *g* for 1 h to recover the final supernatant.

The level of MDA was assessed using the Sigma assay kit (MAK085) according to the protocol of Ohkawa et al. [62]. The content of GSH and SOD activity was computed depending on the assays of Beutler et al. [63] and Velkova-Jordanoska et al. [64]. The GSH was estimated at 412 nm using 5,5′-dithio-bis-2-nitrobenzoic acid in the supernatant fraction. The level of SOD was calculated using the xanthine oxidase–cytochrome protocol using a spectrophotometer at 505 nm. Xanthine interacted with 2-[4-iodophenyl]-3-[4-nitrophenyl]-5-phenyl-tetrazolium chloride (INT) to compose superoxide radicals producing red-colored formazan. This product was utilized to measure the activity of SOD as SOD conjugate with superoxide radicals and consequently controls the formazan synthesis.

The activity of CAT was monitored depending on the decrease in hydrogen peroxide (H_2_O_2_) at 240 nm using a light plate and a spectrophotometer with 1.0 mL quartz cuvettes according to the method of Aksenes and Njaa [65].

### 5.11. Histopathological Investigation

Samples from the liver and kidneys were gathered from all investigated groups, fixed using 10% buffered neutral formalin, then exposed to dehydration in ascending degrees of alcohol, cleared using xylene, and soaked in paraffin. Paraffin sections of about 5 μm in thickness were arranged and stained using hematoxylin and eosin (H&E) and then inspected by an optical microscope, depending on the protocol of Suvarna et al. [66].

### 5.12. Determination of Pb Residues in Fish Muscles

After terminating the experiment (45 days), representative samples were dissected from the dorsal muscle of each group and dried in an oven at 85 °C until they reached a stabilized weight. The prepared samples were weighed (1 g dry weight) and placed in a muffle furnace (Shelton, CT, USA) for 6 h of ashing. After the procedure outlined by Golberg et al. [67], the samples were digested using 5 mL of freshly made perchloric acid (HCLO_4_; 70%) and nitric acid (HNO_3_; 65% *v*/*v*) to Teflon beakers and heating at 50 °C for approximately 5 h to completely break down the organic matter. The digested solution was chilled at ~21 ± 2 °C and diluted using deionized water to reach a final volume of 50 mL. The atomic absorption spectrophotometer was used to analyze each sample separately for calculating the Pb ion residues [68].

### 5.13. Data Analysis

The Shapiro–Wilk test was first conducted to evaluate whether all the data were normal. To determine whether there was a statistically significant difference between treatments, a one-way analysis of variance (ANOVA) was performed with Tukey’s post hoc analysis (SPSS version 18; IBM Corp., Armonk, NY, USA). The Kaplan–Meier protocol was used to analyze survival according to Kaplan and Meier [69]. A *p*-value of less than 0.05 represents statistical variance, including all tests.

## Figures and Tables

**Table 1:** Liver and kidney function biomarkers of *C. gariepinus* exposed to magnetite nanogel (MNG) and/or lead (Pb) as a water exposure for 45 days (*n* = 12/group).

Parameters	Control	MNG	Pb	MNG + Pb
ALT (U L^−1^)	16.33 ± 0.93 ^c^	17.75 ± 1.91 ^c^	25.08 ± 1.17 ^a^	20.25 ± 1.23 ^b^
AST (U L^−1^)	44.95 ± 1.22 ^c^	46.70 ± 0.85 ^c^	94.33 ± 2.20 ^a^	82.58 ± 1.68 ^b^
ALP (U L^−1^)	34.24 ± 1.08 ^c^	34.88 ± 1.35 ^c^	50.20 ± 1.53 ^a^	41.12 ± 0.62 ^b^
Urea (mg dL^−1^)	1.44 ± 0.05 ^c^	1.56 ± 0.04 ^c^	2.75 ± 0.10 ^a^	2.21 ± 0.05 ^b^
Creatinine (mg dL^−1^)	0.27 ± 0.02 ^b^	0.30 ± 0.03 ^b^	0.49 ± 0.50 ^a^	0.34 ± 0.01 ^b^

ALT, alanine aminotransferase; AST, aspartate aminotransferase; ALP, alkaline phosphatase. Values (means ± SE) in the same row that did not have the same superscripts differ significantly (one-way ANOVA; *p* < 0.05).

**Table 2:** Hepatic oxidant/antioxidant biomarkers of *C. gariepinus* exposed to magnetite nanogel (MNG) and/or lead (Pb) as a water exposure for 45 days (*n* = 12/group).

Parameters	Control	MNG	Pb	MNG + Pb
MDA (nmol mg^−1^)	0.64 ± 0.15 ^c^	0.99 ± 0.05 ^c^	11.55 ± 0.58 ^a^	3.06 ± 0.32 ^b^
GSH (ng mg^−1^)	113.57 ± 1.84 ^b^	143.76 ± 2.42 ^a^	41.21 ± 0.43 ^d^	71.69 ± 1.19 ^c^
SOD (U mg^−1^)	88.23 ± 1.79 ^b^	157.67 ± 3.55 ^a^	12.73 ± 0.49 ^d^	62.82 ± 1.31 ^c^
CAT (ng mg^−1^)	22.20 ± 0.57 ^b^	47.30 ± 1.65 ^a^	4.91 ± 0.19 ^d^	8.79 ± 0.15 ^c^

MDA, malondialdehyde; GSH, reduced glutathione content; SOD, superoxide dismutase; CAT, catalase. Values (means ± SE) in the same row that did not have the same superscripts differ significantly (one-way ANOVA; *p* < 0.05).

**Table 3:** Mortality and clinical observations of *C. gariepinus* exposed to different concentrations of magnetite nanogel (MNG) for 15 days.

Conc. (mg L^−1^)	Mortality (*n* = 10)	Clinical Observations		
		Erratic Swimming	Loss of Escape Reflex	External Symptoms (Hemorrhages, Darkness, Fin Rot, and Ulcerations)
0.0	0/10	-	-	-
0.2	0/10	-	-	-
0.4	0/10	-	-	-
0.6	0/10	-	-	-
0.8	0/10	-	-	-
1	0/10	-	-	-
1.2	0/10	-	-	-
1.4	0/10	-	-	-

(-) No abnormal observations.

## Data Availability

The datasets generated or analyzed during the current study are not publicly available but are available from the corresponding author upon reasonable request.
